# Regional evidence of modulation of cardiac adiponectin level in dilated cardiomyopathy: pilot study in a porcine animal model

**DOI:** 10.1186/1475-2840-11-143

**Published:** 2012-11-19

**Authors:** Chiara Caselli, Vincenzo Lionetti, Manuela Cabiati, Tommaso Prescimone, Giovanni D Aquaro, Virginia Ottaviano, Fabio Bernini, Letizia Mattii, Silvia Del Ry, Daniela Giannessi

**Affiliations:** 1 Consiglio Nazionale delle Ricerche (CNR), Institute of Clinical Physiology, Laboratory of Cardiovascular Biochemistry, Pisa, Italy; 2 Department of Medicine, Scuola Superiore Sant’Anna, Pisa, Italy; 3 Fondazione CNR-Regione Toscana “G. Monasterio”, Pisa, Italy; 4 Department of Experimental Pathology BMIE, Faculty of Medicine, University of Pisa, Pisa, Italy; 5 Human Section of Histology and Medical Embryology, Department of Human Morphology and Applied Biology, University of Pisa, Pisa, Italy

**Keywords:** Adiponectin receptors, Heart failure, Animal models, AMPK, Adiponectin

## Abstract

**Background:**

The role of systemic and myocardial adiponectin (ADN) in dilated cardiomyopathy is still debated. We tested the regulation of both systemic and myocardial ADN and the relationship with AMP-activated protein kinase (AMPK) activity in a swine model of non-ischemic dilated cardiomyopathy.

**Methods and results:**

Cardiac tissue was collected from seven instrumented adult male minipigs by pacing the left ventricular (LV) free wall (180 beats/min, 3 weeks), both from pacing (PS) and opposite sites (OS), and from five controls. Circulating ADN levels were inversely related to global and regional cardiac function. Myocardial ADN in PS was down-regulated compared to control (p < 0.05), yet ADN receptor 1 was significantly up-regulated (p < 0.05). No modifications of AMPK were observed in either region of the failing heart. Similarly, myocardial mRNA levels of PPARγ, PPARα, TNFα, iNOS were unchanged compared to controls.

**Conclusions:**

Paradoxically, circulating ADN did not show any cardioprotective effect, confirming its role as negative prognostic biomarker of heart failure. Myocardial ADN was reduced in PS compared to control in an AMPK-independent fashion, suggesting the occurrence of novel mechanisms by which reduced cardiac ADN levels may regionally mediate the decline of cardiac function.

## Background

Adiponectin (ADN) is an adipocyte-derived protein that is abundantly present in plasma [1] and is also expressed in cardiomyocytes [2] and endothelial cells [3]. Two ADN receptors, AdipoR1 and AdipoR2, have been characterized: AdipoR1 is abundantly expressed in skeletal muscle, whereas AdipoR2 is predominantly expressed in the liver [4]. T-cadherin, an additional ADN receptor, is expressed in cardiomyocytes and is involved in ADN-mediated cardioprotection [5]. The remodeling of the failing heart has induced a number of investigators to test the hypothesis that modulation of myocardial metabolism might prove therapeutically advantageous [6].

Potential downstream effectors of ADN receptors include AMP-activated protein kinase (AMPK) and peroxisome proliferator-activated receptor-α (PPAR-α). AMPK is considered an energy-sensing enzyme that on stimulation enhances glucose use and fatty acid oxidation, whereas PPAR-α is a key nuclear transcription factor, regulating expression of genes involved in fatty acid uptake and use [7]. It was recently shown that cardiac ADN and its receptors are elicited by PPAR-γ activation, confirming the role of ADN in maintaining myocardial energy homeostasis [2].

In addition, cardioprotective effects of ADN have been well-demonstrated [8-11]. Many clinical studies have shown that ADN is inversely correlated with an increased cardiovascular risk, and hypo-adiponectinemia has been established as an independent cardiovascular risk factor in coronary artery disease [12-17]. Similarly, in patients with dilated cardiomyopathy (DCM) without overt heart failure (HF), ADN down-regulation is associated with severe endothelial/microvascular dysfunction [18]. Conversely, in patients with overt HF, ADN levels are increased [19] in presence of an increased mortality risk [20]. Further investigation of the autocrine/paracrine role of the cardiac ADN system in the failing heart at different stages could aid in understanding the “ADN paradox” [21]. This issue is still debated and little information is available.

The main aim of our study was to assess the regional modulation of ADN system in a clinically relevant model of non-ischemic HF, both at cardiac and systemic circulating levels. For this purpose, the myocardial expression of ADN, ADN receptors and modulators was determined in an experimental model of LV-pacing induced HF [22] and compared with healthy myocardium. ADN signaling was investigated to address myocardial remodeling, inflammation and metabolic process in different regions of the non-ischemic failing left ventricle (LV) regarding biochemical and functional patterns.

## Methods

### Experimental animal protocol

A total of twelve male adult minipigs (30–40 kg bw) were studied. Non-ischemic HF was induced in seven animals by epicardial pacing of the left ventricle (LV) at 180 beats/min for 3 weeks. Minipigs were sedated with a cocktail of tiletamine hydrochloride and zolazepam hydrochloride (8 mg/kg im) and premedicated with atropine sulfate (0.1 mg/kg im). General anesthesia was subsequently induced with propofol (2–4 mg/kg iv) and maintained with 1–2% isoflurane in 60% air and 40% oxygen. A thoracotomy was performed in the left fifth intercostal space, then a catheter was inserted in the descending thoracic aorta and a solid-state pressure gauge (Konigsberg Instruments) inserted in the left ventricle (LV) through the apex. A Doppler flow probe (Craig Hartley) was placed around the left anterior descending coronary artery, and a screw-type unipolar, epicardial pacing lead (5071 IS-1 UNI; Medtronic, Inc) was attached to the LV free wall, approximately 3 cm distal to the atrioventricular margin. A programmable pacemaker (PREVAILTM, Medtronic, Inc) was implanted in a subcutaneous pocket. Baseline measurements were taken after 7–10 days of post-surgical recovery. Pigs were considered to be in severe HF when left ventricular end-diastolic pressure was ≥ 20 mmHg. Hemodynamic and magnetic resonance imaging (MRI, 1.5 Tesla) measurements were performed at baseline and after 21 days of pacing in sedated animals as previously described [22,23]. The minipigs were finally sacrificed by injecting saturated KCl solution intravenously after deep sedation with 3.3 mg/kg of propofol. The heart was quickly removed and cardiac tissue samples immediately placed in ice-cold RNAlater (Qiagen, Germany) and formalin solution, neutral buffered, 10% (Sigma-Aldrich, St. Louis, MO, USA) stored at −80°C. For our analysis, we selected LV regions surrounding the site of pacing, i.e., the anterior and anterior-lateral regions, called pacing site (PS), and remote from the pacing site, i.e., the inferior and septal-inferior regions, called opposite site (OS). Five healthy minipigs were also studied as controls. Before and after the procedure, peripheral blood samples were withdrawn from animals in tubes containing EDTA (1 mg/mL) and plasma samples were stored at −20°C in aliquots. Animal instrumentation and experimental protocols were approved by the Animal Care Committee according to Italian legislation, following the National Institute of Health publication *Guide for Care and Use of Laboratory Animals*.

### Cardiac MRI measurements

Cine-MRI images were acquired with a 1.5 Tesla MRI scanner (Signa Excite HD, GE Medical Systems, Waukesha, WI, USA) in sedated animals with continuous infusion of midazolam (0.1 mg·kg-1·h iv) at spontaneous heart rate. As previously described by us and by others [23,24] global (end-diastolic volume, end-systolic volume, and ejection fraction) LV parameters of contractile function were analyzed in pacing-induced HF with a commercially available research software package (Mass Analysis, Leyden, The Netherlands). To regionally assess the presence of tissue fibrosis, gadolinium-delayed contrast-enhanced images were acquired in two-dimensional segmented inversion recovery-prepared gradient echo sequence 10 min after administration of contrast agent Gd-DTPA (0.2 mmol/kg iv) in short-axis views.

### RNA and protein extraction

Total RNA and proteins were extracted from LV samples by TRI-REAGENT (MRC, Inc, Cincinnati, OH, USA), as previously described [25]. Total RNA was purified by RNeasy Kit and DNase I (Qiagen, Germany). The extracted RNA was solubilized in RNase-free water and quantified spectophotometrically at 260 nm. The ratio of O.D. values at 260 nm and 280 nm provided an estimate of RNA purity. For each sample, the RNA integrity was evaluated by electrophoresis in a 1.5% agarose gel. The proteins extracted from the organic phase were quantified by Lowry protein assay, using bovine serum albumin as standard.

### mRNA expression analysis (RT-PCR)

For each sample, 5 μg of total RNA were reverse transcribed to cDNA by iScript cDNA Synthesis Kit (Biorad, Hercules, CA, USA) in 100 μL of total reaction volume, according to the manufacturer’s instructions. The expression of ADN, AdipoR1, AdipoR2, T-cadherin, brain natriuretic peptides (BNP), tumor necrosis factor (TNF)-α, inducible nitric oxide synthase (iNOS), were performed using specific primers and conditions (Table 1). All PCR reactions were performed in a 20-μl total volume, containing 0.5 U of *Taq* polimerase (Qiagen, Germany), deoxyribonucleotide triphosphate (dNTP) 0.4 mM, MgCl2 1.5 mM and forward and reverse primers, 200 nM each. To avoid the errors related to the common practice of using only one control gene, the three most stable reference genes selected from different abundance and functional classes in porcine cardiac tissue [26], glyceraldehyde 3-phosphate dehydrogenase (GAPDH), hypoxanthine phosphoribosyltransferase 1 (HPRT-1) and TATA binding protein (TBP), were evaluated using specific primers and specific PCR conditions (Table 1) and their geometric mean was used as normalization factor [27]. PCR products were separated on 1.5% agarose gel and stained with GelStarNucleic Acid Gel Stain (Cambrex East Rutherford, NJ, USA). Pictures of resulting gels were taken with digital camera and image analysis was performed by QuantityOne Software (Biorad).

**Table 1:** Sequence of RT-PCR primer pairs for analyzed genes

**Primer**	**GenBank**	**Sequence**	**Ta, °C**	**Cycles, n**
ADN	EF601160.1	Forward: ATCTGGAGTGACTGGGGTTG	61	36
*202 bp*		Reverse: TTTGCCAGTGGTGACATCAT		
AdipoR1	AB527058.1	Forward: AACCCACCCAAAGCTGAAGA	58	29
*334 bp*		Reverse: CTGAGCATGGTCAAGATTCC		
AdipoR2	NM_001007192	Forward: GCCTGGGGATCTTTTATATGTTTC	53	34
*650 bp*		Reverse: GCCGATCATGAAACGAA		
T-CAD	NM_001109945.1	Forward: CCCGGGCAGAGCTTCGAAAT	64	25
*217 bp*		Reverse: GGTAGAAAACCGGGCCCTCG		
PPAR-α	NM_001044526.1	Forward: TCGCGGGAAAGGCCAGCAAT	70	27
*431 bp*		Reverse: GTCGTCCAGCTCCAGCGCATT		
PPAR-γ	DQ437884.1	Forward: AGGAGCATAGCAAAGAGGTGGCC	70	32
*391 bp*		Reverse: AGCAAACCTGGGCGGTCTCCA		
BNP	M25547	Forward: GTGCTCCTGCTCCTGTTCTT	61	35
*451 bp*		Reverse: TCCCAGGCTTCTGTGAGG		
iNOS	NM_001143690.1	Forward: GAGGGCAGCCAAGGCCCAAG	65	35
*264 bp*		Reverse: GGCCAGCTTTCGGAGCAGCA		
TNF-α	EU682384.1	Forward: ATCGGCCCCCAGAAGGAAGAC	64	33
*351 bp*		Reverse: GATGGCAGAGAGGAGGTTGAC		
GAPDH	AF017079	Forward: ACCACAGTCCATGCCATCAC	60	30
*451 bp*		Reverse: TCCACCACCCTGTTGCTGTA		
HPRT1	DQ178126	Forward: CCGAGGATTTGGAAAAGGT	60	30
*181 bp*		Reverse: CTATTTCTGTTCAGTGCTTTGATGT		
TBP	DQ178129	Forward: GATGGACGTTCGGTTTAGG	60	30
*124 bp*		Reverse: AGCAGCACAGTACGAGCAA		

### Western blotting

Total proteins obtained from cardiac tissue or plasma samples were subjected to western blot analysis after sodium dodecyl sulfate-polyacrylamide gel electrophoresis (SDS-PAGE), under reducing and denaturating conditions. After boiling at 95°C for 5 min, samples (70 μg of total protein or 2 μL of plasma per lane) were subjected to SDS-PAGE on 10% polyacrylamide gel and then transferred to polyvinylidene fluoride (PVDF) membrane for ADN and nitrocellulose membrane for phospho-AMPKα and AMPKα. Nonspecific binding was blocked by incubation with 5% nonfat dry milk for 3 h. The membranes were incubated overnight at 4°C with primary antibody against ADN (1:2000, Millipore, Billerica, MA, USA); AMPKα (1:1000, Cell Signaling Technologies, Boston, MA, USA) and phospho-AMPKα(1:1000, Cell Signaling Thecnologies). After washing, the membranes were incubated with anti-rabbit biotinilated (1:20000, Enzo Life Sciences International, Plymouth Meeting, PA, USA) or anti-mouse biotinilated (1:10000, Enzo Life Sciences International) for 1 h at room temperature and then, to amplify the signal, with horseradish peroxidase-conjugated streptavidin (1:2000, Biorad) for 30 min at room temperature. Sample loading was normalized by immunoblotting with anti-tubulin polyclonal antibody (1:500, Serotec, Oxford, UK). Proteins were visualized by colorimetric reaction using opti-4CN, (Biorad) and analyzed using QuantityOne Software (Biorad).

### Histochemistry and immunohistochemistry

Formalin-fixed tissue specimens were embedded in paraffin. Five-micron-thick sections were cut and serially mounted on glasses. Sections were stained with hematoxilin and eosin stain and van Gieson’s counterstain, to detect myocardial collagen deposits. Immunohistochemical analysis was performed to detect ADN. Briefly, sections were treated as follows: microwave antigen retrieval (700 W for 7 min, twice in 10 mM sodium citrate, pH 6.0); hydrogen peroxide (3% in PBS 1X) to block endogenous peroxidase and normal goat serum (10% in PBS 1X) to block non-specific bindings. ADN was detected by incubation overnight at 4 °C with the primary antibody (1:500, Millipore). Antibodies were diluted in PBS containing 0.1% goat serum and 0.1% TRITON. Sections were sequentially incubated with biotinylated anti-rabbit anti-serum (1:500, Enzo Life Sciences International), horseradish peroxidase–streptavidin complex (1:1000, Biorad), 3,3¢-diaminobenzidine tetrahydrocloride (DAB). Specimens were counterstained with hematoxylin (1:30), mounted with DPXÒ mountant for histology (Fluka, Buchs, Switzerland) and examined by light microscope. Negative controls were obtained by using secondary goat serum (1:100).

### Circulating and cardiac TNF-α measurement

Arterial blood samples were collected during the imaging acquisition. Plasma levels as well as myocardial concentrations of porcine TNF-α were measured in duplicate by a specific ELISA kit (Thermo Scientific/Pierce, Rockford, IL, USA), with an assay range of 31.3–2000 pg/mL and a sensitivity < 5.0 pg/mL.

### Statistical analysis

Data are reported as media ± SEM. Statistical comparisons were performed between PS, OS and controls by statistical software (SPSS 16.0 for Mac, Chicago, IL, USA). Differences between groups were compared by ANOVA followed by the Fisher post hoc test. A two-tailed P-value < 0.05 was considered statistically significant.

## Results

### Global and regional LV function

Hemodynamic and MRI parameters of HF animals are reported in Table 2. All animals displayed the typical alterations of HF, including significant reduction of LV ejection fraction (LVEF) compared to healthy animals and increase of LV end-diastolic pressure (LVEDP), which reached 20 ± 6 mmHg after 21 ± 2 days of pacing. This is considered a stage of severe, although not terminal, HF [22].

**Table 2:** Global and regional LV function

	**Baseline**	**3 weeks of LV pacing**
Heart Rate, bpm	94±6	122±8*
MAP, mmHg	109±8	77±8.4*
LVEDP, mmHg	5.5±3.04	20.5±6*
LV dP/dtmax , mmHg/s	2400±220	1388±188*
LV EDV, ml	65±5	85±7.2*
LV ESV, ml	15±3.4	54.4±7.6*
LV EF, %	76.9±2	36±1.8*
LV ESWT (PS), %	63±3.2	5±1.2*
LV ESWT (OS), %	62±2.5	40±1.8*

Values are means±SE. MAP, mean arterial pressure; LVEDP, left ventricular end diastolic pressure; EDV, end diastolic volume; ESV, end systolic volume; EF, ejection fraction; ESWT end systolic wall thickening; PS, pacing site; OS, opposte site. n= 7.

* vs Baseline, p<0.05.

### Circulating and cardiac ADN

**Figure 1:**
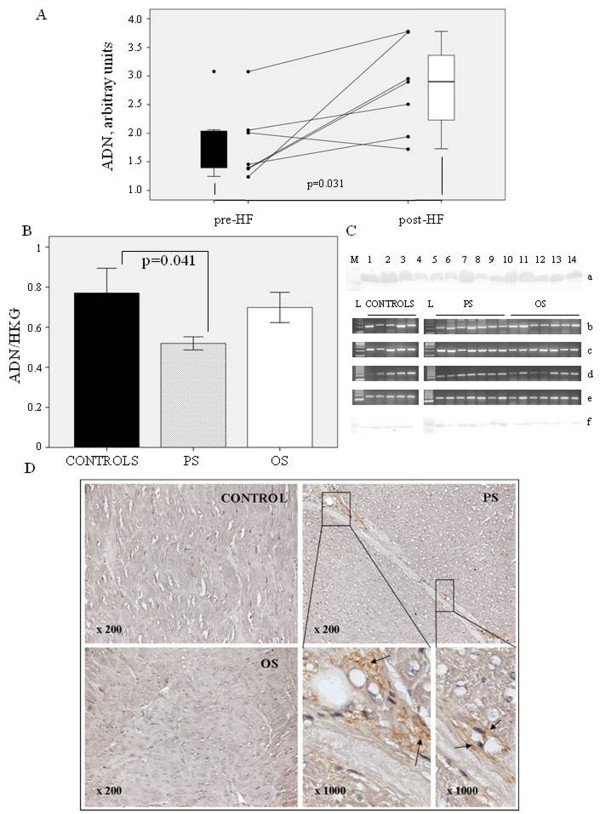
**ADN presence in both plasma and cardiac tissue. A**) Circulating levels of ADN measured by Western Blotting before the beginning of the experimental protocol and after 21 days of pacing induction (n = 7); **B**) ADN expression at mRNA level from PS, OS and controls; **C**) relative blots of circulating levels of ADN (a); mRNA expression of ADN (b) as well as housekeeping genes, GAPDH (c), HPRT-1 (d), TBP (e) measured by RT-PCR and immunoblots of ADN by Western blot (f); **D**) representative immunostaining of ADN from heart sections showing the extra-cellular localization of ADN in peri-vascular tissue of PS.

Circulating ADN levels measured by Western blotting increased significantly (p = 0.031) in pigs after 21 days of pacing (Figure 1A).

The presence of ADN in cardiac muscle was checked by RT-PCR, western blotting and immunohistochemistry. ADN mRNA expression was downregulated in HF cardiac samples (Figure 1B), and it was significantly lower in PS compared to controls (p = 0.041). As showed in Figure 1C, myocardial ADN protein expression was very low. Similarly, ADN was weakly detected by immunohistochemistry in extracellular matrix surrounding vessel walls of PS, whereas no signal was detected in histological section of LV samples from OS and healthy animals (Figure 1D).

### ADN receptors

**Figure 2:**
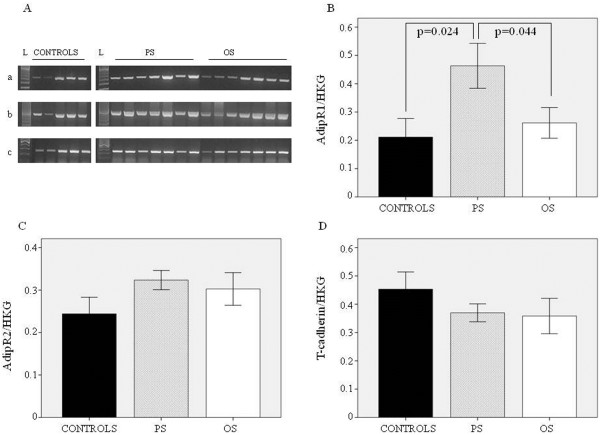
**ADN receptors in cardiac tissue from both PS and OS as well as in control hearts. A**) relative blots of AdipoR1 (a), AdipoR2 (b) and T-cadherin (c) RT-PCR products; **B**) mRNA expression of Adipo R1; **C**) AdipoR2, and **D**) T-cadherin.

mRNA expression of ADN receptors in cardiac muscle was determined by RT-PCR (Figure 2). As expected, in porcine heart AdipoR1 was expressed more than AdipoR2, as shown by the lower number of PCR cycles used to obtain the right number of cDNA copies in RT-PCR analysis (Table 1). AdipoR1 was significantly upregulated in PS when compared to OS (p = 0.044) and controls (p = 0.024) and showed a negative correlation with ADN mRNA expression (r = −0.717, p = 0.001). Myocardial AdipoR2 and T-cadherin mRNA expression in failing heart was unchanged compared to control. A positive correlation between the AdipoRs was observed (r = 0.582, p = 0.009).

### ADN regulation

**Figure 3:**
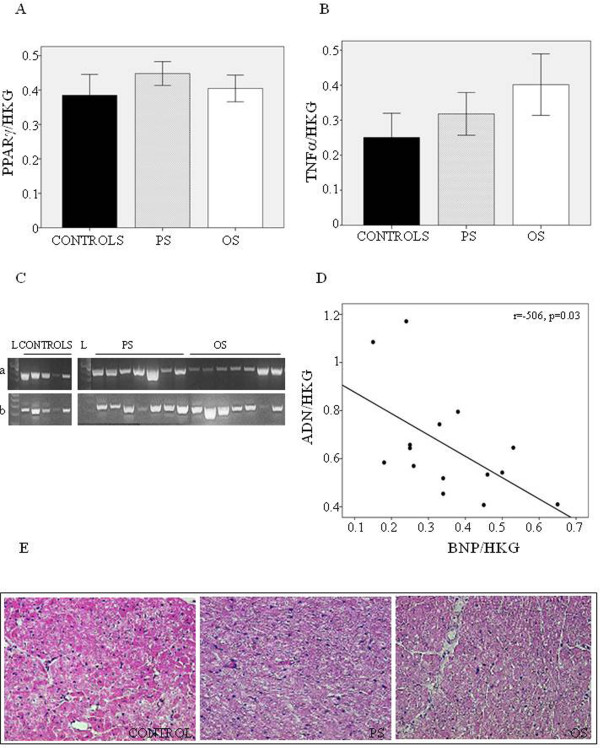
**ADN regulation. A**) mRNA expression of PPARγ and **B**) TNFα in PS, OS and controls **C**)as well as relative blots of RT-PCR products **D**); relationships between ADN and BNP mRNA expression; **E**) representative immunostaining of hematoxilin and eosin stain from heart sections.

To understand the mechanism underlying ADN system modification, mRNA expression of PPARγ, an upstream ADN regulator [8], was determined in each region, but no changes compared to control were observed in PS and OS (Figure 3A). To investigate the relationship between the inflammatory process and ADN level in cardiac tissue, TNF-α expression was measured in both cardiac tissue and peripheral circulation. Myocardial TNF-α level in HF animals did not show any significant change compared to controls at both mRNA (Figure 3B) and protein expression (7.82 ± 2.04 pg/mg of total protein in PS, 8.85 ± 2.36 in OS, 8.53 ± 2.40 in controls, p = ns). TNF-α was not detected in peripheral circulation either at baseline or at 21 days of sustained pacing. No inflammatory cells were detected in either region of the failing heart as nor in controls (Figure 3E), confirming the lack of inflammation. Finally, we found that ADN is inversely correlated with BNP (P < 0.05) (Figure 3D).

### ADN signaling

**Figure 4:**
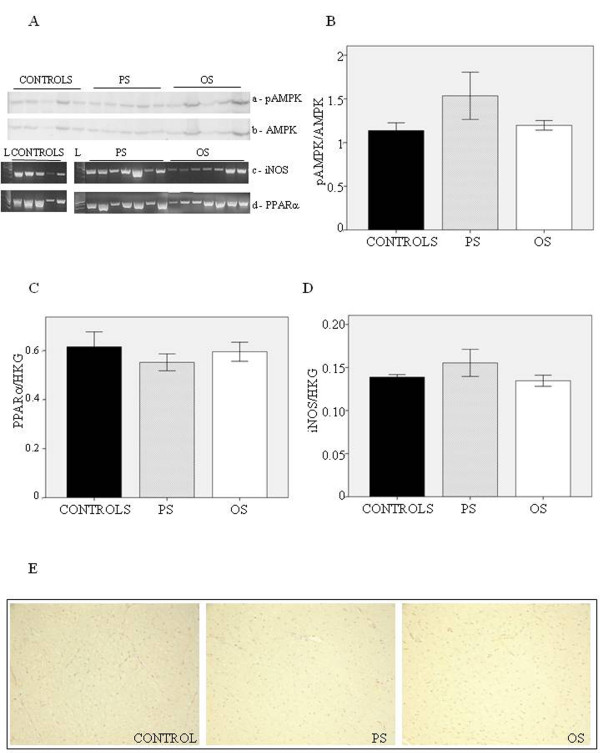
**Cardiac molecular pathways involved in ADN signaling. A**) relative immunoblots of pAMPK and AMPK as well as mRNA expression of PPARα and iNOS; **B**) the ratio of phospho-AMPK and AMPK in PS, OS and controls; **C**) mRNA expression of PPARα; **D**) mRNA expression of iNOS; **E**) van Gieson’s staining of heart section from PS, OS and controls.

Figure 4A shows the results of RT-PCR and WB analysis performed in healthy and failing hearts. To explore the relationship between ADN and metabolism as well as apoptotic pathways, the regional activation of AMPKα and PPARα expression were investigated. As shown in Figure 4B, the myocardial expression and phosphorylation of AMPKα showed no changes in failing hearts compared to controls. In addition, the expression of PPARα (Figure 4C) and iNOS in HF PS and OS was equal to that in controls.

### Cardiac remodeling

Histological measures in HF PS and OS revealed no difference in collagen deposits compared to controls, yet a weak positive stain was detected in the perivascular area of the pacing site (Figure 4E), confirming the absence of gross myocardial fibrosis in presence of severe regional contractile failure [22].

## Discussion

We investigated the myocardial ADN pathway in a clinically relevant animal model of non-ischemic dilated cardiomyopathy. The main findings of our study are: first, increased circulating ADN levels in HF minipigs did not affect myocardial AMPK expression and activity, nor PPARα expression; second, sustained high-rate LV pacing caused a significant reduction of ADN level and increased AdipoR1 level in PS, but not in OS; third, regional ADN down-regulation was related to severe contractile impairment, yet the key regulators of altered cardiac metabolism, inflammation and remodeling were unchanged compared to control hearts.

High circulating ADN levels have been reported repeatedly in HF [19-21,28] and several mechanisms have been utilized to explain this increase. Recent studies have reported that natriuretic peptides enhance ADN production via the cGMP pathway, by human adipocytes both *in vitro* and *in vivo* in patients with HF [29,30]. We have previously described that BNP and CNP expression increased in adult minipigs after 21 days of LV pacing [25]. It is well-known that natriuretic peptides are secreted almost exclusively by cardiac tissue and their levels are increased in patients with HF [31]. It has been hypothesized that the ADN increase could be a compensatory effect in order to restore metabolic homeostasis, due to its anti-inflammatory and insulin-sensitizing effects [32,33]. Abnormalities in glucose regulation, such as impaired glucose tolerance and insulin resistance, have been shown to correlate with HF severity [34]. In our animal model, we previously found that plasma insulin concentration was significantly higher in HF compared to control in presence of homogenous LV increase of glucose uptake [22]. In our study, no change was observed in AMPK activity and PPARα expression in presence of a marked reduction of global and regional LV function. The lack of cardiac activation of the ADN downstream molecules could suggest that their associated cardiac effects are not related to high ADN plasma levels. This may agree with previous studies reporting an association between high levels of ADN in HF and severity as well as increased mortality, indicating for ADN a role as prognostic biomarker of HF [20,21]. Based on these findings, a “functional ADN resistance” in HF at the receptor level has been hypothesized. This resistance could determine an attenuated ADN response during the progression of HF [21], as recently observed at the level of skeletal muscle in chronic HF [35]. In this paper, a strong positive correlation between AdipoR1 and PPARα/AMPK gene expression was found. In our study, we found an increased expression of AdipoR1 in the HF pacing site in presence of myocardial ADN mRNA down-regulation, yet PPARα/AMPK gene expression and AMPK activity were not significantly different from healthy heart. As to T-cadherin, a physical association of ADN with T-cadherin is a prerequisite for ADN’s physiological activity in the heart [5]; thus, the reduction of T-cadherin mRNA expression in failing heart, although not significant, could support the reduced activity of ADN/AdipoR system in this condition.

The different behavior of systemic and cardiac ADN expression (Figure 1) as well as the presence of a regional variation in myocardial tissue after pacing are in tune with the existence of a local cardiac ADN regulation, independent of the circulating ADN, as previously described in human [36,37] and murine hearts [38]. The cardiac protein expression of ADN in our samples was difficult to detect. We detected small amounts of ADN in the extracellular matrix surrounding the injured areas of the PS, but not in OS and controls. Consistent with these findings, previous studies have reported the presence of ADN in damaged regions of the heart, also due to its leakage from the vascular section [39-41]. Moreover, down-regulation of ADN mRNA expression, compared to controls, and the myocardial over-expression of AdipoR1 in PS suggested the existence of a possible feedback loop, as previously observed in skeletal muscle [35].

The mechanisms underlying this down-regulation are yet unknown. It is well-recognized that inflammatory cytokine production, particularly TNF-α, plays a critical pathogenic role in cardiovascular complications, and a reciprocal action between TNF-α and ADN exists [42,43]. However, in our model we found that cardiac ADN was down-regulated in absence of inflammatory response. In fact, TNF-α at the mRNA and protein level, was unchanged in each region of failing left ventricle and no inflammatory cells were detected. Accordingly, myocardial expression of PPAR-γ, a known upstream regulator of ADN [2], was also unchanged in each region of the failing heart. On the other hand, Sturk et al. provided evidence, directly in cardiomyocytes, of a cardiac regulatory feedback loop in ADN expression, without identifying any specific cardiomyocyte-derived secreted factors that exert this negative feedback [36]. Otherwise, a negative relationship between ADN and BNP mRNA expression was observed in this study (Figure 3C), suggesting that impaired contractile function in the failing heart could affect the ADN/AdipoR1 system. In a recent study in murine hearts with transverse aortic constriction, inverse correlations between myocardial ADN and BNP as well as heparin-binding epidermal growth factor (HB-EGF) were shown, indicating an important role for ADN in mediating the myocardial hypertrophic signaling pathway [38]. However, in our experimental animal model, cardiac tissue did not develop hypertrophic remodeling, as shown by the collagen immunohistochemistry (Figure 3E). The lack of collagen deposits in PS in absence of coronary stenosis and inflammation suggests the occurrence of non-ischemic ventricular remodeling due to mechanical stress, which could impair the cardiac ADN/AdipoR1 system. Sen *et al.*[44] demonstrated in vitro that continuous mechanical strain inhibits ADN expression at the transcriptional level.

As a matter of fact, it has been suggested that ADN may directly protect cardiomyocytes [37] largely via an AMPK-mediated signal pathway [9]. However, the lack of variation of AMPK expression and activity in our animal model supports the hypothesis that other pathways drive the cardioprotection afforded by ADN. In fact, treatment with ADN is still effective in improving the cardiac function in AMPK knockout mice [45]. The cardioprotective action of ADN may be performed by suppression of TNF-α signaling via the COX-2-prostaglandin E2-linked cascade [46]. In our conditions, TNF-α did not show any changes between HF and controls either at mRNA or protein level (Figure 4). In addition, it has been reported that ADN might prevent excess NO generation by inhibiting iNOS expression [45]. In our model, no significant variation of iNOS expression was found in HF compared to controls and no correlation with myocardial ADN was observed.

### Limitations

Other downstream ADN signalling pathways such as ceramides [47,48] and other factors besides ADN that could affect the transduction signalling [49,50] exist; however, the samples collected in our study are inconsistent for their evaluation. The main limitation of this study is the impossibility of evaluating these further mechanisms involved in both ADN and AMPK signalling.

## Conclusions

Our findings were observed in a reliable translational animal model of non-ischemic HF. The pacing-induced HF model is considered the gold standard in HF experimental research due to its relative similarities with many features of clinical dilated cardiomyopathy [51]. In conclusion, we observed that in spite of its high peripheral concentrations, plasma ADN did not show cardioprotective effects, confirming its role as a negative prognostic biomarker of HF. In particular, the effect of adiponectin in non-ischemic HF is AMPK-independent. These observations suggest the occurrence of novel mechanisms by which reduced cardiac ADN levels may regionally mediate the decline of cardiac function.

## Abbreviations

AdipoR1: Adiponectin receptor 1; AdipoR2: Adiponectin receptor 2; ADN: Adiponectin; AMPK: AMP-activated protein kinase; BNP: Brain natriuretic peptides; DCM: Dilated cardiomiopathy; GAPDH: Glyceraldehyde 3-phosphate dehydrogenase; HF: Heart failure; HPRT-1: Hypoxanthine phosphoribosyltransferase 1; HKG: Housekeeping gene; iNOS: Inducible nitric oxide synthase; LV: Left ventricle; LVEDP: Left ventricle diastolic pressure; LVEF: Left ventricle ejection fraction; OS: Opposite site; PPAR-α: Peroxisome proliferator-activated receptor α; PPAR-γ: Peroxisome proliferator-activated receptor γ; PS: Pacing site; RT-PCR: Reverse trascriptase polymerase chain reaction; TBP: TATA binding protein; T-CAD: T-cadherin; TNF-α: Tumor necrosis factor α.

## Competing interests

The authors declare they have no competing interests.

## Authors’ contributions

As to the contribution of each author, CC and DG designed the study, analyzed and interpreted the results, and drafted the manuscript; GDA and FB were involved in the animal model; MC, TP and VO made substantial contributions to performing the experimental protocol; VL, LM and SDR were involved in revising the manuscript critically for important intellectual content. All authors participated in the discussion and interpretation of the results and in the final approval of the manuscript submitted.
